# First record of gregarines (Apicomplexa) in seminal vesicle of insect

**DOI:** 10.1038/s41598-017-00289-3

**Published:** 2017-03-14

**Authors:** Glenda Dias, Romano Dallai, Antonio Carapelli, João P. P. Almeida, Lucio A. O. Campos, Leda R. A. Faroni, José Lino-Neto

**Affiliations:** 1 Departamento de Biologia Geral, Universidade Federal de Viçosa, 36570-900 Viçosa, Minas Gerais Brazil; 2 Dipartimento Scienze della Vita, Università degli Studi di Siena, Via Aldo Moro 2, 53100 Siena, Italy; 3 Departamento de Engenharia Agrícola, Universidade Federal de Viçosa, 36570-900 Viçosa, Minas Gerais Brazil

## Abstract

Gregarines (Apicomplexa) are a diverse group of protozoan parasites, which infects gut and other body cavities of invertebrate hosts. In reproductive system of insects, gregarine has been reported only in the accessory glands and spermathecae of females; therefore, this is the first report of a gregarine species in seminal vesicles of insects. Different developmental stages, including sporozoytes, oocysts and trophozoites were described from morphological descriptions using light and electron transmission microscopy. The parasites were described in seminal vesicles of the beetle *Tribolium castaneum* a model organism and an important insect pest. DNA sequence analysis suggests that the protozoan parasite was an *Ascogregarina* sp.

## Introduction

Gregarines are a heterogeneous group of Apicomplexan protozoan parasites comprising about 1,600 species. Mature forms consist of large and extracellular parasites, typically infecting a variety of invertebrates, especially annelids, arthropods and molluscs^1–6^. These parasites have received great attention from many researchers, including those interested in the parasite-host coevolution as well as those interested in biological control of insects^8–10^.

The gregarine life cycles include the following stages: sporozoites (cell form that infects new hosts); trophozoites or gamonts (large extracellular vegetative stages); gametocyst (gamont pairs in which gametes are produced); and oocyst (it is a hardy, thick-walled spore, which contains the infective stages)^11^. In general, the contamination by gregarine occurs via faecal-oral transmission, when the parasites enter the body by oocyst ingestion containing several sporozoites. Then the sporozoites reach the intestinal cavity, attach to the host cells, and develop extra-cellular into larger vegetative stages^11^.

Among insect, Orthoptera, Odonata, Blattodea, Diptera, and Coleoptera have been reported to be infected by gregarine^1, 4, 5, 10^. The presence of these parasites is recorded especially in digestive tracts of larvae and adults, Malpighian tubules, fat bodies and eggs^10^. In the reproductive system of insects, gregarines were reported only in accessory glands and spermathecae of females^9, 10, 12^. As part of an ongoing study of spermiogenesis in the stored grain pest, *Tribolium castaneum* (Coleoptera: Tenebrionidae), we report the novel occurrence of a gregarine species in seminal vesicles of insects.

## Results

**Figure 1:**
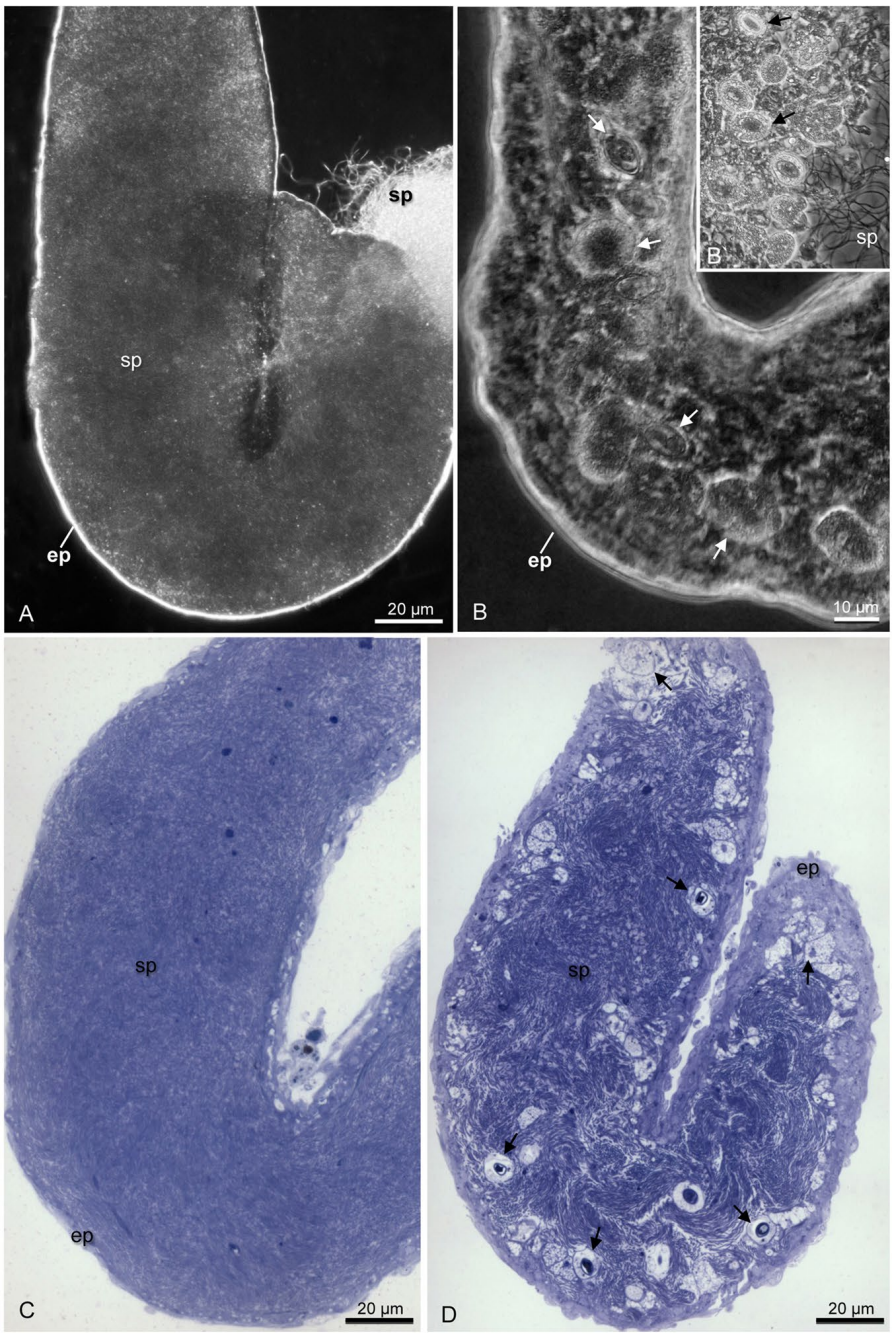
Photomicrographs of seminal vesicles of *Tribolium castaneum* in phase contrast **(A,B,B’**) and histological sections (**C,D**). (**A**) Uncontaminated seminal vesicle in total mount. Spermatozoa (sp); epithelium (ep). (**B,B’**) Contaminated seminal vesicle in total mount. The arrows indicate the parasites. Note in (**B’**) the large amount of parasites from seminal vesicle broken. (**C**) Histological section of uncontaminated seminal vesicle. Observe the lumen completely filled with only sperm (sp). (**D**) Histological section of contaminated seminal vesicle. Note the parasites in different stages (arrows) and located preferentially in the peripheral region.

The seminal vesicles of sexually mature *Tribolium castaneum* are characterized by a dilatation of the deferent duct, filled with spermatozoa (Fig. 1A,C). Seminal vesicles of all sampled individuals of the contaminated colony showed parasites (Fig. 1B,D). Under the light microscope, the uncontaminated seminal vesicles in whole mount exhibited a smooth surface (Fig. 1A), while those infected reflects the presence of high contamination showing an uneven surface (Fig. 1B). When the contaminated seminal vesicles were disrupted a large amount of parasites were released together with spermatozoa (Fig. 1B).

**Figure 2:**
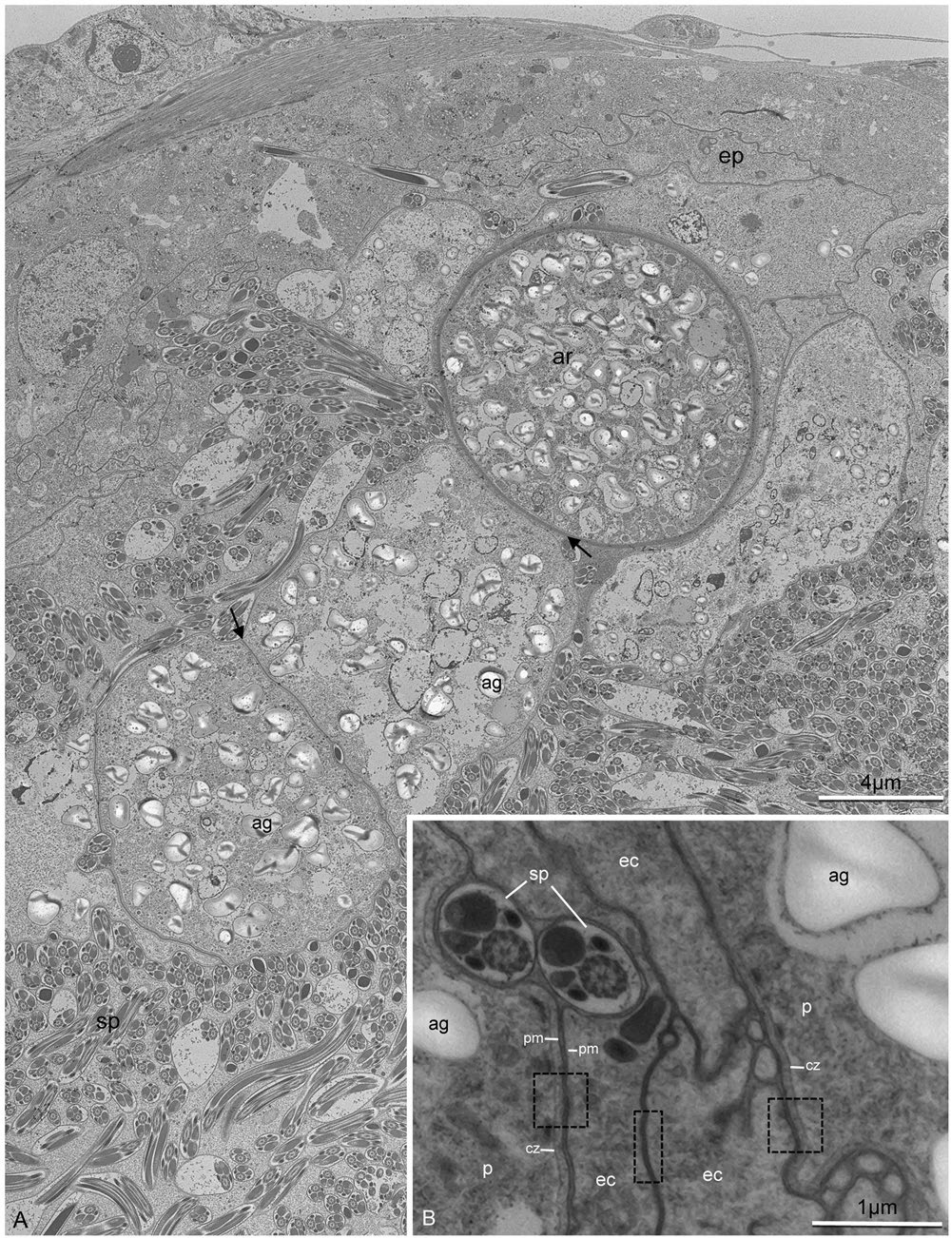
Electron micrographs of contaminated seminal vesicle of *T. castaneum*. (**A**) Segmented trophozoite with the anterior region (ar) inserted into the seminal vesicle epithelium (ep). Note the many amylopectin granules (ag) in all segments (arrows). Spermatozoa (sp). (**B**) Intimate association between the plasma membrane (pm) of the parasite (p) and plasma membrane of epithelial cells (ec). Note the junction between two epithelial cells (rectangle), and junctions between epithelial and parasitic membranes (squares). Cortical zone of the parasite (cz).

**Figure 3:**
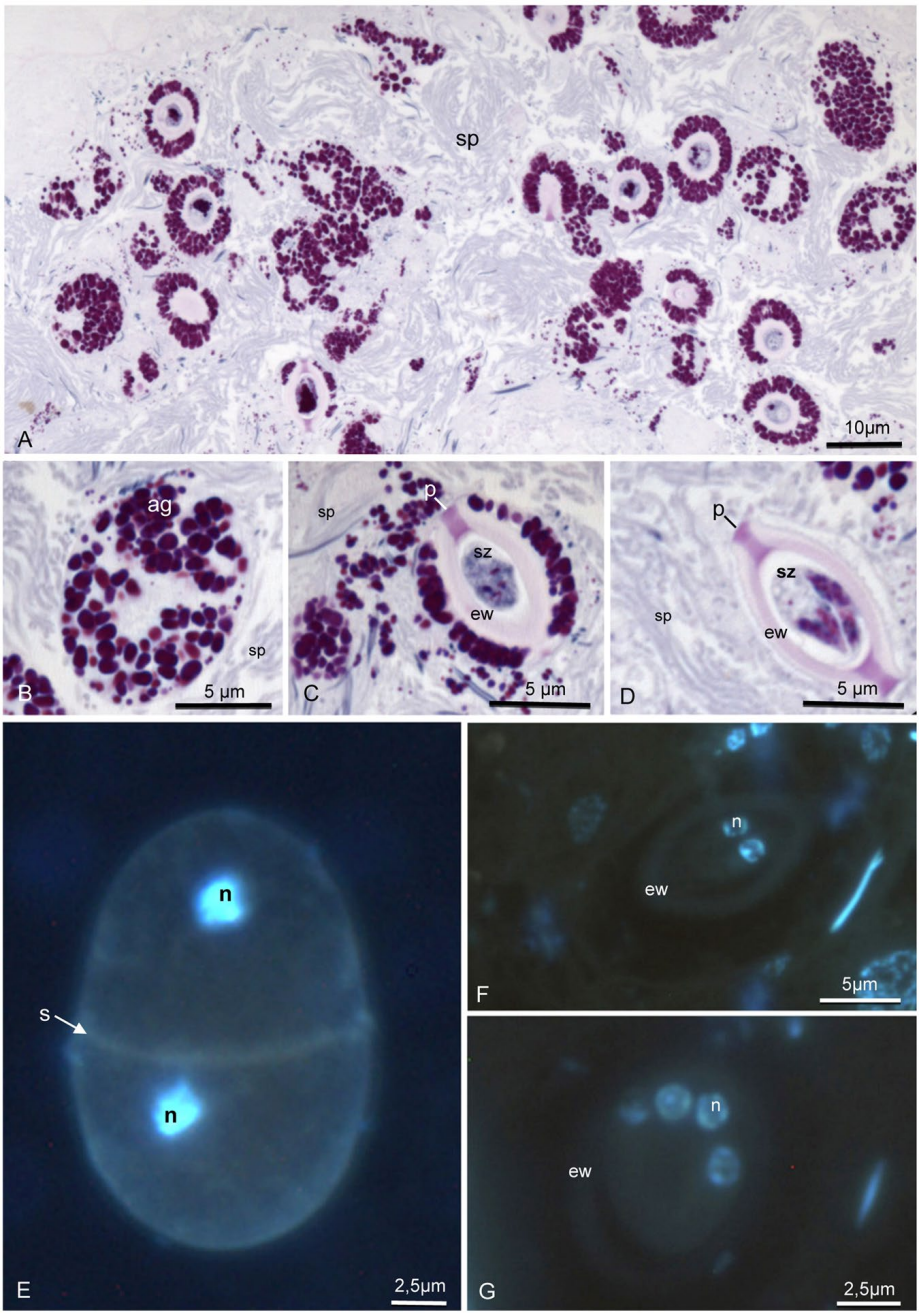
Histological section of contaminated seminal vesicle submitted to periodic acid-Schiff (PAS) reaction (**A–D**), and parasites stained with DAPI (**E–G**). (**A–D**) Gregarines in different stages exhibiting amylopectin granules (ag) in strong purple color. Note in oocytes (**C,D**) the presence of plugs (p) on the both poles and internally the sporozoites (sz). Spermatozoa (sp); extracellular wall (ew). (**E**) Gamont stage; Note the presence of two nuclei (n) and a septum (s). (**F,G**) Oocytes showing two (**F**) and four (**G**) sporozoites surrounded by a thick wall (ew).

**Figure 4:**
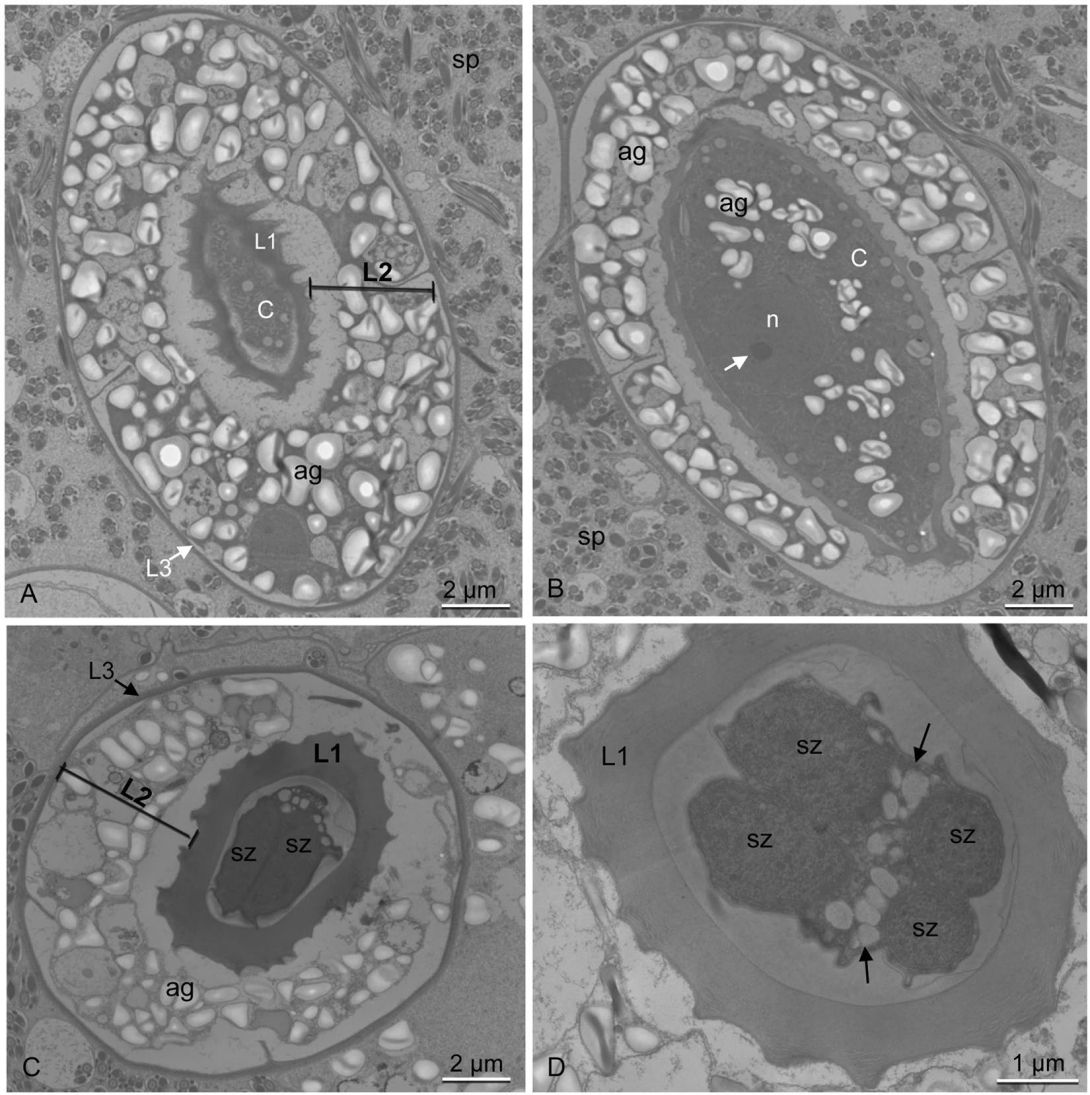
Electron micrographs of oocytes. (**A,B**) Sagittal section of oocytes showing the thick extracellular wall with three layers (L1–L3). In (**B**) note a single sporozoite (sp) showing nucleus (n) and small nucleolus (arrow). Cytoplasm (c); amylopectin granules (ag); spermatozoa (sp). (**C,D**) Transverse section of oocytes showing two (**C**) and four (**D**) sporozoites (sz) linked by cytoplasmic bridges (arrows).

Histological sections of the contaminated seminal vesicles showed parasites preferably located close to the vesicular epithelium (Fig. 1D), which was formed by a simple layer of cubic cells. In the uncontaminated seminal vesicles lumen had only spermatozoa (Fig. 1C) and the epithelium was formed by flattened cells. The parasites occurred as single cells with different morphologies, which may represent distinct stages of the life cycle (Fig. 1B,D). The trophozoites were observed closely associated to the epithelial cells of the seminal vesicles (Fig. 2A,B). Under high resolution micrograph observed an intimate association of the trophozoite membrane to the membrane of the epithelial cells (Fig. 2B). In this stage they were large, about 30 µm, segmented and with irregular shape. They have cytoplasm filled with large amounts of amylopectin granules (Fig. 2A,B), which showed a strong purple color in the PAS tests (Fig. 3A). The gametocyst had two juxtaposed cells (trophozoites or gamontes) surrounded by a thin wall, with a septum between them (Fig. 3E). In this stage there was also a great amount of amylopectin granules (Fig. 3A). The nucleus of the each gamont was evident with DAPI staining preparation (Fig. 3E) as well as those of the sporozoites within the oocysts (Fig. 3F,G). The oocysts were individualized and exhibit shape very uniform (Figs 3B,C,D and 4A,B). They were barrel shape, about 10 µm in length, and exhibited plugs on the both poles, which were also PAS positive (Fig. 3C,D). The cells showed a thick wall, about 2,5 µm, with three layers: the inner, was formed for numerous longitudinal lamellae extending from one pole to another, with a smooth surface associated with the cell membrane, and the opposite side had indentations; the median layer was thicker, with less electrondensity and amorphous with its larger area filled by amylopectin granules and; the outer layer thiner, flat and electrondense (Fig. 4A–D). The oocyst wall enclosed one or more sporozoites (Figs 3F,G and 4C,D), linked by cytoplasmic bridges (Fig. 4D). The sporozoites had nucleus with decondensed chromatin and evident nucleolus, and also amylopectin granules in the cytoplasm (Fig. 4B).

**Figure 5:**
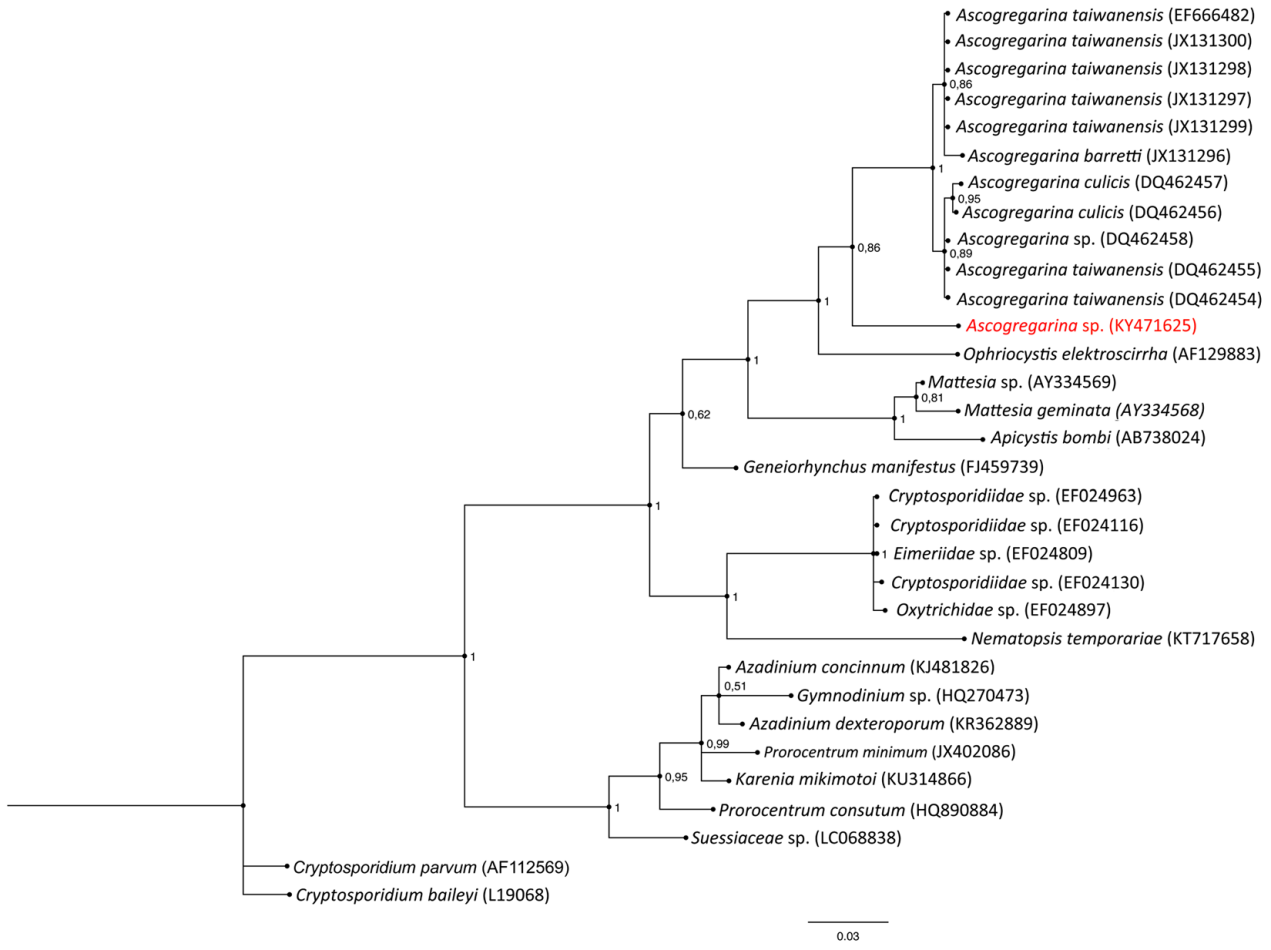
Bayesian tree inferred from partial SSU (small subunit) rDNA sequences showing phylogenetic position of the gregarine from *T. castaneum*. *Cryptosporidium* genus sequences were used as outgroup. Codes in parentheses represent GenBank accession numbers of partial SSU rDNA sequences. Posterior Probabilities (PP) values are shown with the nodes.

The gregarine DNA fragment was amplified only from the two extractions obtained from infected seminal vesicles, whereas no amplification of the parasite DNA resulted from either non-infected vesicles and whole beetles deprived of the reproductive system. Similarly to the results of Dabert and Dabert^13^, the PCR reaction revealed two bands for the extractions from the seminal vesicles, of approximately 1 and 3 kb, respectively. Following the reference paper, we hypothesized that the primer couple, used for the PCR reaction, was unable to exclusively match the protozoan DNA, and that host/parasite selection was only possible by observing the size differences of co-amplified products, as revealed by electrophoresis. In this respect, in agreement with Dabert and Dabert^13^ results, the larger band was amplified from the host rDNA, whereas the smaller was obtained from the parasite extraction. Therefore, the two PCR products were separated, excising the targeted band, after a short run on a standard electrophoresis gel. DNA recover from the gel was performed using the kit Wizard SV Gel and PCR Clean-up (Promega, Madison, WI, USA). Purified 1 kb was initially sequenced with both PCR primers, obtaining a clear reading of electropherograms only for f1300. In order to obtain the required double-strand reading of the PCR product, two species-specific primers (Api-18Sf and Api-18Sr) were designed on the sequence obtained with f1300, and than used for additional sequencing reactions. Final consensus rDNA sequences (662 bp in length), encompassing the 18 S and ITS2 rDNA of the parasites DNA present on the seminal vesicles of two *T. castaneum* specimens, were obtained and deposited in GenBank, under accession numbers KY471625 and KY471626. Among the first ten sequences producing significant alignments with the query retrieved in Blastn search, all matches identified three species (barretti, culicis and taiwanensis) of the *Ascogregarina* genus, with a query cover range between 72–73%, E-value = 0 and 92–93% of nucleotide identity. Phylogenetic analysis (Fig. 5) revealed that our sequence forms a well supported cluster (PP = 1) with all *Ascogregarina* sequences present in GenBank. Phylogenetic position inside the genus is uncertain due to low support obtained in this analysis (PP = 0.86).

## Discussion

The seminal vesicles of *Tribolium castaneum*, as in most insects, are dilations of the vasa deferentia, in which sperm are stored before they are transferred to the female^14, 15^. The vesicular epithelium, in several animals including the insects, has glandular that produce nutrients for nourishment and maintenance of the sperm^16, 17^. This makes the seminal vesicles a suitable environment for parasites, as has been observed in studies with earthworms^7, 18^. However, the presence of parasites in the seminal vesicles of insects has not been observed so far, therefore this is the first study that provides the record of gregarine apicomplexan parasites in this reproductive organ. The infection process by gregarine in seminal vesicles of *T. castaneum* probably occurs with a similar mechanism as described by Lantova and Volf^10^ for the accessory glands of the female of sand flies. In this study it was reported that the gregarines enter into the intestinal cavity through ingestion, go through the intestinal wall and reach the accessory gland, where they adhere to the wall and finally are released into the gland lumen. The occurrence of intestinal gregarine has already been recorded for several insects, including the tenebrionid beetles^4, 19^, and the contamination process is known, which occurs through ingestion of oocysts during feeding^10, 20^. In the earthworm seminal vesicles the life cycle and infection process were well established and showed that the contamination occurred by an oro-fecal route, with the parasite crossing the intestinal wall to reach the dorsal vessel and the heart from where it was transported to the seminal vesicles^21^.

We obtained high-resolution electron transmission micrographs showing also an intimate association between the membrane of parasites and the epithelial cells of the seminal vesicle, suggesting an adhesion mechanism similar to that described by Cox^22^. As known, the apicomplexan members are characterized by the presence of an organelle called apical complex^23^. This structure is a complex assemblage of structural and secretory elements at the apical point of the cell required to in the host cell invasion process and nutrition^24^. However in eugregarine species, for example, this structure is lost during trophozoite development stage suggesting a different mode of nutrition to the group. According Cox^22^ the cortex folds of eugregarine trophozoites are likely structural adaptations that create the surface area necessary to effectively absorb nutrients passing through the host intestinal tract.

Trophozoites are the most structurally diverse stages in gregarine life cycles and their ultrastructural traits have been used as a taxonomic too^13, 20^. The mature stage is characterized by shape unsegmented or subdivided into distinct regions and the cytoplasm usually appears filled with large amounts of amylopectin granules, which were confirmed from the PAS tests. In this study the trophozoites are segmented and a great number of these granules was also observed in all the other developmental stages, except for sporozoites. Some studies suggest that the role of amylopectines is essential for gametogenesis due to gametocyst wall formation and supplying of energy to the parasite. The oocysts observed in the seminal vesicles of *T. castaneum* are characterized by oval-shaped and contained a thick resistant wall. They exhibit plugs on the poles, which possibly are open for in order to release the sporozoites^25^.

The impact of gregarine infection on host fitness and viability is widely discussed. According Valigurová^23^, although gregarines are not lethal to the insect hosts, they reduce longevity, fecundity and body size of the host. High levels of infection by gregarines in the digestive epithelium of insects can cause some defects to development or damage the host tissue^26^. This pathogenicity is mainly attributed to trophozoites, which may destroy individual cells through their embedded epimerites or cause significant impact on the host nutritional state by obstruction of the gut^27^. On the other hand, gregarines are relatively harmless to their hosts and some of them even consider that they are essential to the well being of the host^23, 26, 28^. According to Sumner^28^ is possible that gregarine secrete essential substances such enzymes or vitamins necessary for larval growth. Others studies have shown that some gregarine species found in mosquito intestines are pathogenic^29, 30^, suggesting them as possible agents for biological control^31^.

Based on morphological characteristics, we supposed that the species belongs to the eugregarine group. DNA sequence analysis in GenBank showed a clear nucleotide similarity between the parasite hosted in the seminal vesicles of *T. castaneum* and three species of the genus *Ascogregarina*, such as *A. barretti, A. culicis* and *A. taiwanensis*, parasites of the midgut of mosquitoes *Ochlerotatus triseriatus*, *Aedes aegypti*, and *Ae. albopictus*, respectively^32^. Despite the uncertainty of the phylogenetic position inside the genus, our bayesian inference showed the close relation of the parasite sequence obtained in this study with the *Ascogregarina* genus. We therefore conclude that the are strong evidences to suggest that the parasite of the seminal vesicle belongs to this genus *Ascogregarina* (Lecudinidae). The fact of the colony has been reared in the laboratory for more than 10 years and with practically 100% of infected males indicates that this infection is widespread among males. Still considering that no amplification of the parasite DNA resulted from whole beetles deprived of the infected seminal vesicles, it is possible that this parasite is specific of seminal vesicle of these beetles. Thus, given the capacity of transmission and supposed specificity, these parasites most likely belong to a new species of *Ascogregarina*. This finding opens a new avenue for further studies regarding the effect of this parasite on the reproductive fitness of the insect with the potential to use it in pest insect control.

## Materials and Methods

Males of *Tribolium castaneum* infected and uninfected by gregarines were obtained from contaminated and uncontaminated colonies maintained at the Laboratório de Manejo Integrado de Pragas de Grãos Armazenados, Departamento de Engenharia Agrícola, Universidade Federal de Viçosa (UFV), in Viçosa, Minas Gerais State, Brazil. To observe the degree of infection 50 males from the contaminated colony and 20 males from the uncontaminated colonies were used.

### Light Microscopy

To observe contaminated and uncontaminated seminal vesicles in whole mount, adult males were dissected and seminal vesicles removed in 0.1 M sodium phosphate buffer solution, pH 7.2, fixed for 1 h in 4% paraformaldehyde in 0.1 M sodium phosphate buffer, pH 7.2 and transferred to histological slides covered with coverslips and photographed using an Olympus BX-60 microscope equipped with phase contrast (Olympus Corporation, Tokyo, Japan).

Some isolated seminal vesicles were dissected out and the parasites and spermatozoa were spread on slides, fixed with solution of 4% paraformaldehyde in 0.1 M sodium phosphate buffer, pH 7.2 for 30 minutes, washed in running water and dried at room temperature. The slides were examined and the parasites were photographed in Olympus BX-60 photomicroscope equipped with phase contrast. To observe the nuclei some slides were stained with 0.2 mg/ml4,6-diamino-2-phenylindole (DAPI).

### Histological Sections

To obtain the histological sections, the contaminated and uncontaminated seminal vesicles were dissected and fixed for 2 h in 2.5% glutaraldehyde solution in 0.1 M phosphate buffer, pH 7.2 at 4 °C. The material was washed for 2 h in the same buffer, post fixed in 1% osmium tetroxide for 2 h and dehydrated in alcohol solutions of increasing concentrations: 30, 50, 70, 90 and 100%. The material was immersed in two 4-h baths each at room temperature, the first with a mixture of historesine (Leica Historesin, Heidelberg, Germany) and alcohol (1:1), and the second with pure historesin. For inclusion, the seminal vesicles were immersed in historesin with a catalyst in silicone moulds, which were placed in Petri dishes and transferred to an oven at 58 °C for 24 h. Semithin sections (2 µm) were obtained with a microtome Leica RM 2155 (Leica Corporation, Wetzlar, Germany) with glass knives. These were transferred to histological slides stained with Harris haematoxylin for 15 min, washed in running water for 10 min, stained with eosin for 1 min and rapidly rinsed in tap water. For the detection of neutral polysaccharides some slides were submitted to the periodic acid-Schiff (PAS) reaction. All observations and photographs were made using an Olympus BX-60 microscope.

### Transmission Electron Microscopy

The contaminated seminal vesicles were dissected in 0.1 M sodium cacodylate buffer, pH 7.2, and fixed in a 2.5% glutaraldehyde solution containing 0.2% picric acid, 3% sucrose and 5 mM CaCl_2_ in 0.1 M sodium cacodylate buffer, pH 7.2, for approximately 24 h at 4 °C. The material was post-fixed in a 1% osmium tetroxide solution for 2 h, dehydrated in an increasing alcohol series, infiltrated and finally embedded in epoxy resin (Epon 812). Ultrathin sections obtained with a Reichert Ultracut II E ultramicrotome, were contrasted with solutions of 3% uranyl acetate of and 0.2% lead citrate and then observed with a Philips CM 10 electron microscope operating at 80 kV (Universitá degli Studio di Siena, Siena–Italy) and a Zeiss EM 109 (Núcleo de Microscopia e Microanálise, Universidade Federal de Viçosa, MG–Brasil).

### DNA extraction and PCR

Total genomic DNA was extracted from: two males of either infested or non-infested *T. castaneum* whole specimens, with the reproductive system removed; and from the seminal vesicles isolated from other two specimens for each infested and non-infested coleopteran hosts. Total DNA of the eight samples was extracted with the Wizard SV genomic DNA purification system kit (Promega, Madison, WI, USA) and used for PCR amplifications. A gregarine-specific DNA fragment, encompassing the 18S rDNA and ITS2 regions, was amplified using the primer pair: f1300 (5′-TGCATGGCCGTTCTTAGTTG-3′) and ITS2-28S (5′-ATATGCTTAAATTCAGGGGG-3′)^13^. The PCR reaction, run in a GeneAmp PCR System 2700 (Applied Biosystems, Foster City, CA, USA) thermal cycler, was performed in a volume of 25 μl containing: 2.5 μl of genomic DNA, 0.5 mM of each primer, 0.2 mM of each dinucleotide, 2.5 mM of MgCl_2_, 5 μl of Green GoTaq Flexi buffer and 0.625 u of GoTaq Flexi DNA Polymerase (Promega, Madison, WI, USA). PCR conditions were: 35 cycles at 95 °C for 1 min, 50 °C for 1 min, and 72 °C for 90 sec, followed by a final extension step at 72 °C for 10 min. PCR products were then purified using the kit Wizard SV Gel and PCR Clean-up (Promega, Madison, WI, USA), and sequenced with the above PCR primers and with other two internal primers, Api-18Sf (5′-GTAATTATTCATCTTGAACGAGGAA-3′) and Api-18Sr (5′-TTCCTCGTTCAAGATGAATAATTAC-3′), specifically designed on the targeted sequence. Sequencing reactions were run on a DNA Analyzer ABI 3730, at the core facility of the Biofab Research Lab (Rome, Italy). The sequence data set was assembled using Sequencher 4.4.2 (Gene Codes Corporation, Ann Arbor, MI, USA).

### Sequence analysis

The sequence obtained was searched for highly similar DNA sequences in the database of nucleotide collection of NCBI using the Blastn tool. For Bayesian phylogenetic inference a dataset containing our sequence and 31 reference sequences from Genbank were aligned using ClustalW^33^ provided in MEGA 6.0^34^ (GenBank accession codes are reported in Fig. 5). To infer the best nucleotide substitution model for the alignment, we used the program MrModelTest 2.3^35^ under the Akaike Information Criterion (AIC). The trees were searched using the software MrBayes 3.2^36^ provided in the webserver CIPRES^37^ with two independent runs, with four Markov chains each (one cold and three heated). Each chain ran for 50,000,000 generations and was sampled every 5,000 generations. A burn-in on the first 25% of the trees was performed before using the remaining topologies to build a consensus topology with its respective branch lengths, which was viewed using FigTree v.1.4.2^38^. Tree was rooted using *Cryptosporidium parvum* (AF112569) and *C. baileyi* (L19068) as outgroup. Statistical support at each node was evaluated by calculating the Posterior Probability (PP).
